# Evaluation of a New Standardized Nasal Sampling Method for Detection of SARS-CoV-2 RNA via RT-PCR

**DOI:** 10.3390/microorganisms12010210

**Published:** 2024-01-20

**Authors:** Johannes G. M. Koeleman, Sander Mol, Henk Brand, David S. Y. Ong

**Affiliations:** 1 Department of Medical Microbiology and Infection Control, Franciscus Gasthuis & Vlietland Hospital, 3045 PM Rotterdam, The Netherlands; h.koeleman@franciscus.nl (J.G.M.K.); h.brand@franciscus.nl (H.B.); 2 Emergency Department, Franciscus Gasthuis & Vlietland Hospital, 3045 PM Rotterdam, The Netherlands; s.mol3@franciscus.nl; 3 Department of Epidemiology, Julius Center for Health Sciences and Primary Care, University Medical Center Utrecht, 3584 CX Utrecht, The Netherlands

**Keywords:** SARS-CoV-2, COVID-19, screening, nasal sampling, Rhinoswab

## Abstract

The aim of this study was to compare the diagnostic accuracy of nasal sampling using a novel anterior nasal swab (ANS) (Rhinoswab) versus combined oro-nasopharyngeal (OP/NP) sampling in COVID-19 suspected patients. This prospective observational study was performed from 11 November to 2 December 2021 (part 1), and from 16 January to 22 February 2022 (part 2). Adult patients who attended the emergency room with suspected COVID-19 were asked to participate. One ANS and one OP/NP sample were consecutively collected, and both were analyzed via reverse transcription polymerase chain reaction (RT-PCR). The result of the OP/NP sample was considered to be the reference standard. A total of 412 patients were included, of whom 171 (41.5%) had a positive RT-PCR of the OP/NP swab, whereas 139 (33.7%) were positive on the ANS sample. The overall diagnostic accuracy for ANS sampling in terms of sensitivity, specificity, positive predictive value, and negative predictive value was 80.7% (95% CI 73.8–86.2), 99.6% (95% CI 97.3–100), 99.3% (95% CI 95.5–100), and 87.9% (95% CI 83.3–91.4), respectively. In conclusion, ANS sampling with the Rhinoswab identified 80.7% of all presented COVID-19 patients in an emergency department. Future studies should investigate if nasal Rhinoswab self-sampling is suitable for reliable diagnosis of COVID-19 in an outpatient setting.

## 1. Introduction

Accurate and rapid identification of coronavirus disease 2019 (COVID-19) patients is essential for optimal patient care, isolation management, and contact tracing to prevent further spread of severe acute respiratory syndrome coronavirus-2 (SARS-CoV-2) infections. SARS-CoV-2 is transmitted via small liquid aerosols or larger respiratory droplets [1]. The binding and infecting of human-ciliated cells to the cellular receptor angiotensin-converting enzyme 2 (ACE2) is facilitated by a receptor-binding domain within the spike protein. Human cells with ACE2 receptors are most frequent in the upper respiratory tract (i.e., nasal cavity, oropharynx, and laryngopharynx) and decreases in frequency further along the distal airways [2,3]. Initial infection of the nasal passage leads to cytopathic destruction of the nasal epithelial cells responsible for nasal mucociliary clearance, and thus, it results in symptoms of a runny nose. Also, there may be an infection of sustentacular or supporting olfactory epithelial cells which can cause anosmia (loss of smell). These two commonly observed initial COVID-19 symptoms support a model of infection beginning in the nasal cavity [4].

The reference standard for SARS-CoV-2 testing is reverse transcription polymerase chain reaction (RT-PCR) targeting the RNA genome of SARS-CoV-2 in upper-respiratory specimens [5,6,7]. Initial diagnostic testing for current COVID-19, as recommended by the World Health Organization (WHO), consists of collecting and testing a nasopharyngeal (NP) specimen [8]. However, NP sampling requires trained healthcare workers, cooperative patients, and the use of specific sampling swabs. In addition, many individuals resist NP swabbing because of the fear of discomfort and pain during this procedure. Therefore, there is increasing interest in less invasive sampling strategies using alternative sample types such as throat, nasal, or saliva samples. These samples can be obtained easier and mostly pain-free, which makes these sampling methods more patient-friendly. Accordingly, such samples can be self-collected by patients with simple instructions.

**Figure 1:**
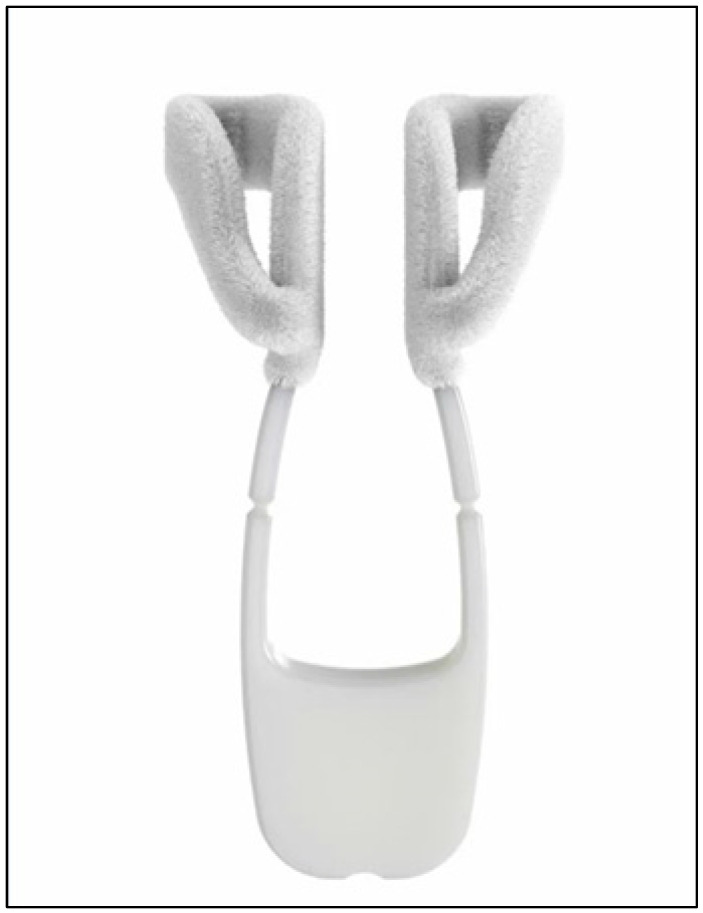
Picture of a Rhinoswab.

Recently, a new noninvasive alternative method for anterior nasal sample (ANS) collection by using a new designed swab (Rhinoswab, Rhinomed, Melbourne, Australia) was developed. This nasal swab consists of a double-loops nylon-flocked swab with large surface areas for simultaneous sampling of both nostrils (Figure 1). To date, the diagnostic performance of this alternative sampling method for SARS-CoV-2 testing in patients presenting to the hospital has not yet been evaluated. The aim of this prospective study was to compare the diagnostic accuracy of the ANS sampling by use of the Rhinoswab to the current hospital standard, i.e., combined oropharyngeal and nasopharyngeal (OP/NP) sampling, in COVID-19 suspected patients. In addition, healthcare worker’s experiences with these two sampling methods were compared.

## 2. Materials and Methods

### 2.1. Study Population and Procedures

The study was performed during two study periods: from 11 November to 2 December 2021 (part 1) and from 16 January to 22 February 2022 (part 2). All adult patients who attended the emergency room with suspected COVID-19 (based on respiratory symptoms), in whom SARS-CoV-2 testing was indicated, were eligible to participate in the study regardless of disease severity or the need for hospital admission. After informed consent was obtained, one ANS and one OP/NP sample were consecutively collected by healthcare workers. The presence of SARS-CoV-2 in the combined OP/NP sample was considered to be the reference standard.

### 2.2. Sampling Methods

ANS samples were obtained by using a Rhinoswab which was inserted into both nostrils until a slight resistance occurred, which were then left in place for 60 s. After this, the swab was either removed immediately (part 1) or after side-to-side movements of the swab for 15 s in the anterior nasal area (so-called extended ANS procedure) (part 2). OP/NP sampling was performed after ANS sampling in order to not contaminate the inside of the nose by the viral material from the nasopharynx. For OP/NP sampling, a flexible mini-tip flocked swab was used and rubbed over the oropharyngeal space besides the uvula and the same swab was placed through one of the nasal passages into the nasopharynx and removed after several rotations. Both samples were placed in separate viral transport media (Mantacc, Miraclean Technology Co, Ltd., Shenzhen City, China), then frozen at −20 °C within 24 h to be stored until further analysis.

### 2.3. Virology Methods

Reverse transcription polymerase chain reaction (RT-PCR) assays were performed using the MagNa Pure96 system and LightCycler 480 II (Roche, Basel, Switzerland), as described by Corman et al. [8]. RNA extraction was performed from clinical samples using the DNA and Viral NA Large Volume kit and subsequently RT-PCR using the Fast Viral Master mix (Life Technologies, Carlsbad, CA, USA). A cycle threshold (Ct) value below 40 was interpreted as positive for SARS-CoV-2 RNA.

### 2.4. Evaluation of Sampling Methods

Trained healthcare workers involved in patient sampling in the emergency department were asked to complete a questionnaire after parts 1 and 2 about their experiences with the Rhinoswab sampling methods regarding the ease of insertion of swabs, apparent patient discomfort, and preference for sampling method. Healthcare workers were asked their professional title (i.e., nurse or medical doctor), to indicate the ease of insertion of Rhinoswabs on a scale of 1 (=very difficult) to 5 (=very easy), to indicate how the patient experienced the sample collection on a scale of 1 (=very uncomfortable) to 5 (=very comfortable), and whether they preferred the OP/NP or the Rhinoswab (i.e., with or without side-to-side movements) sampling method.

### 2.5. Statistical Analysis

The primary outcome was the sensitivity, specificity, positive predictive value (PPV), and negative predictive value (NPV) of the RT-PCR ANS compared with the reference standard of OP/NP. For analysis based on Ct values, the results were expressed as median with an interquartile range (IQR). Groups were compared by using the Mann–Whitney U test for continuous variables and the chi-square test or Fisher’s exact test for categorical variables as appropriate. Correlation between RT-PCR Ct-values of OP/NP and ANS samples was analyzed using Pearson’s correlation coefficient. Values of *p* that were <0.05 were considered to be statistically significant. All data were analyzed using Microsoft Excel version 16, GraphPad Prism version 8, and R version 3.3.2 (R Foundation for Statistical Computing).

### 2.6. Ethical Statement

The study protocol was approved by both the Medical Research Ethics Committee United (protocol number R W21.203) and the Institutional Review Board of FG&V (protocol number 2021-093). These institutions waved the need for written informed consent and agreed to include only documented verbal informed consent in the patient medical records. This study was performed in line with the principles of the Declaration of Helsinki.

## 3. Results

Of the 412 patients included, 171 (41.5%) patients had a positive RT-PCR on the OP/NP swab, whereas 139 (33.7%) were positive according to the ANS sample (Table 1). Both sampling methods were positive in 138 (33.5%) patients and both were negative in 240 (58.3%). In total, 172 (41.7%) patients had a positive result of one or both swabs.

**Figure 2:**
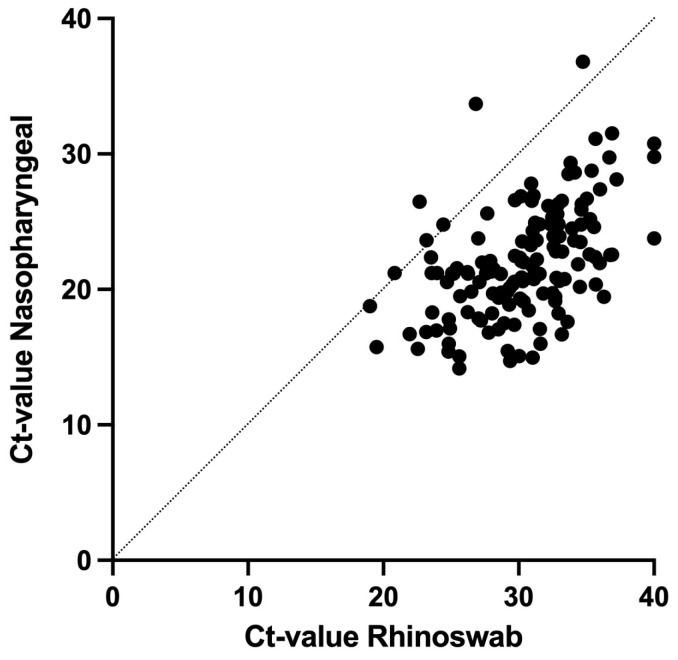
Correlation of SARS-CoV-2 Ct values in combined oro-nasopharyngeal samples and anterior nasal samples.

When the paired OP/NP and ANS samples were compared in patients who tested positive for both, the median Ct for SARS-CoV-2 was significantly different: Ct 21.3 (IQR 19.3–24.5) in OP/NP samples vs. Ct 30.4 (IQR 27.4–33.0) for ANS samples (*p* < 0.01). The Ct scores, as determined by both methods in these concordant PCR positives, were significantly correlated (Pearson’s correlation coefficient 0.50, *p* < 0.01) and showed a lower yield from the ANS swabs (Figure 2). For the 33 (8.0%) discordant samples, of which only the OP/NP sample was positive, the median Ct score was 27.7 (IQR 23.8–29.9).

The overall diagnostic accuracy for ANS in terms of sensitivity, specificity, PPV, and NPV was 80.7% (95% CI 73.8–86.2), 99.6% (95% CI 97.3–100), 99.3% (95% CI 95.5–100), and 87.9% (95% CI 83.3–91.4), respectively. In addition, subgroup analysis of patients with and without the ANS extended procedure showed no statistical differences (*p* = 0.22) in diagnostic sensitivity. To examine the impact of viral load on diagnostic performance, sensitivity rates of ANS were also calculated based on selected positive OP/NP specimens with RT-PCR Ct values of <30, <25, and <20, and an increasing sensitivity of the ANS sampling method of 84.3%, 91.7%, and 95.8%, respectively, was observed.

In total, 80 of 118 (67.8%) healthcare workers completed a survey about their experiences with the ANS sampling method. Of these, 79 were evaluable, of which 72 (91%) were completed by emergency room nurses. Almost two-thirds (65.8%) of the respondents found the Rhinoswab insertion very easy. Furthermore, 33/41 (80.5%) indicated that the ANS sampling method was experienced as comfortable by the patients, while 21/38 (55.3%) indicated this was true for the extended ANS sampling. Most of the healthcare workers preferred ANS sampling (72.2%) compared to OP/NP (17.7%). The respondents’ overall assessment of the different sampling methods of OP/NP, ANS, and extended ANS on a ten-point scoring scale was 5.5, 8.1, and 7.4, respectively.

## 4. Discussion

In our prospective study including 412 emergency room patients suspected for COVID-19, ANS sampling by use of the Rhinoswab was able to detect SARS-CoV-2 RNA in 80.7% as compared to our hospital reference method of combined OP/NP sampling. Sensitivity of this ANS sampling method increased in patient samples with high viral loads (Ct < 20) to above 95%. However, our findings suggest that ANS sampling cannot rule out SARS-CoV-2 infection in unselected patients presenting to an emergency department.

To our knowledge, this is the first comparative study in adults in which this new ANS sampling method was evaluated for diagnostic accuracy for the detection of SARS-CoV-2. A recent study in children showed that the diagnostic accuracy of ANS sampling using Rhinoswab for the detection of respiratory viruses was comparable to a combined throat–nose swab [9]. However, comparison between their and our results remains difficult because the use of a different reference method (i.e., throat–nose versus OP/NP sampling) and only a small portion of samples were positive for SARS-CoV-2 RNA in their study. Several other studies have evaluated the performance of nasal sampling for detection of SARS-CoV-2. Herein, differences in nasal sampling sensitivity ranging from 68% to 96% were reported when compared to nasopharyngeal sampling [10,11,12]. The sensitivity of 80.7% found in our study falls within this reported range. Since we used a more sensitive reference standard, namely a combined OP/NP sample from two different locations, we cannot rule out whether the sensitivity of the new ANS sampling method would be higher when compared to only nasopharyngeal sampling as the reference standard. Therefore, comparing studies of different sampling techniques is complex as it is affected by wide variations in study populations, sample collection, processing protocols, and the use of different reference methods.

In our study, ANS sampling was performed using the Rhinoswab sampling technique. With this standardized method, both nostrils can be sampled simultaneously for a defined period of time in order to collect sufficient nasal secretions and thus optimize the sensitivity of this sampling technique. Nevertheless, in our study, the ANS-sampling method detected fewer SARS-CoV-2 positive cases in comparison to OP/NP sampling. This difference could be explained by the fact that patients presenting to the emergency department may already have a longer duration of illness, and subsequently, most SARS-CoV-2 RNA has already been (partially) cleared in the nose. Indeed, the viral load is expected to be higher when measured during the earliest phase of SARS-CoV-2 infection before the disease progresses further and hospital admission is required. Thus, the difference in diagnostic performance between OP/NP and ANS sampling in the community setting could be less pronounced. Our study showed that in ANS samples with a lower Ct value, which corresponds to a higher viral load, sensitivity increased to above 95%. Another explanation for this could be the fact that we compared the ANS-sampling method with our hospital reference method that sampled both the oropharynx and the nasopharynx. The current WHO recommendation for screening individuals for SARS-CoV-2 infection includes testing a nasopharyngeal sample only [5]. It is certainly plausible that our hospital reference method is more sensitive than the recommended nasopharyngeal sample only.

This study was performed in a time period during which, consecutively, the delta variant and the omicron BA.1 variant were predominant in the Netherlands. Meanwhile, multiple newer Omicron subvariants have emerged that are more immune-evasive in comparison to prior variants, which result in higher transmissibility and higher viral loads. Therefore, it is possible that the sensitivity of the Rhinoswab ANS sampling technique is higher in the context of the current circulating SARS-CoV-2 variants.

In our study, the professional’s experience with the Rhinoswab sampling for ANS collection compared to OP/NP sampling was evaluated. Nasal swab sampling has advantages over NP sampling because it is easier to collect and causes less pain and discomfort to patients. Our study shows this is also true for Rhinoswab collection, since the user survey showed that 66% of the ANS samplers experienced the Rhinoswab insertion as simple, with 80% of them scoring a favorable patient tolerability during ANS sampling.

Since the pandemic phase has passed, COVID-19 is considered to be endemic. Governments have downregulated infection control strategies. The fear for complications of the disease has largely disappeared, which has consequently led to decreasing willingness to test for SARS-CoV-2 infection when having acute respiratory symptoms. The burden of testing and willingness for home isolation, hygiene measures, and wearing masks has become higher for the broader community. Nevertheless, COVID-19 remains to have a large impact, and the number of infections seem to periodically increase. Patients with significant comorbidities, including those with immunocompromised conditions, remain at risk for hospitalization and a more severe disease course. Therefore, the availability to have a more comfortable and reliable sampling method could contribute to curb infection dynamics in the community when needed. Most self-tests require the use of the tip of a swab in the anterior nasal cavity. It remains to be investigated how the diagnostic performance of Rhinoswab using the double-loops nylon-flocked swab with larger surface areas for both nostrils compares to the more widely used nasal swabs in the community setting when applied as self-tests.

This study has several limitations. First, the study was performed in a patient population in a hospital setting where trained healthcare professionals were involved in patient sampling. Therefore, self-collection of Rhinoswab ANS sampling could not be evaluated. Second, the included patients in the emergency room were generally too sick to self-score patient discomfort and pain during both sampling procedures. Thus, the score was entirely determined by the assessment of the healthcare worker. Finally, in this study we have not included the costs of the different sampling methods. However, based on the overall costs associated with hospital care, the pre-analytical and molecular diagnostic costs, which are similar for the different sampling methods, the difference in material costs of the different swabs represents only a very small proportion of the total costs and would be negligible. This could be different when the comparison of sampling methods is applied in a different setting, such as community-based self-testing.

## 5. Conclusions

Testing for SARS-CoV-2 remains important for adequate diagnostic and public health disease control strategies. In this study, we showed that ANS sampling is less sensitive than OP/NP sampling for the identification of hospitalized COVID-19 patients. However, given the benefits of an easier and more patient-friendly procedure, it warrants future studies to assess if nasal Rhinoswab self-sampling is suitable for reliable diagnosis of COVID-19 outside the hospital setting during the early phase of infection.

## Figures and Tables

**Table 1:** Comparison of characteristics of combined oro-nasopharyngeal versus nasal sampling for SARS-CoV-2 detection by RT-PCR.

		Oro-Nasopharyngeal Sampling ^3^						
		Positive	Negative	Total	Sensitivity % (95% CI)	Specificity % (95% CI)	PPV % (95% CI)	NPV % (95% CI)
ANS without extension ^1^	Positive	69	0	69				
	Negative	12	113	125				
	Total	81	113	194	85.2 (72.5–91.8)	100 (95.9–100)	100 (93.4–100)	90.4 (83.4–94.7)
ANS with extension ^2^	Positive	69	1	70				
	Negative	21	127	148				
	Total	90	128	218	76.7 (66.3–84.7)	99.2 (95.1–100)	98.6 (91.2–99.9)	85.8 (78.9–90.8)
ANS total	Positive	138	1	139				
	Negative	33	240	273				
	Total	171	241	412	80.7 (73.8–86.2)	99.6 (97.3–100)	99.3 (95.5–100)	87.9 (83.3–91.4)

^1^ ANS: anterior nasal swab (ANS) sampling with use of Rhinoswab. ^2^ ANS with extension: anterior nasal swab sampling with extended Rhinoswab procedure (see text). ^3^ Oro-nasopharyngeal sampling was the reference standard. PPV: positive predictive value; NPV: negative predictive value.

## Data Availability

The datasets generated during and/or analyzed during the current study are not publicly available but are available from the corresponding author on reasonable request.

## References

[1] Schröder I. (2020). COVID-19: A risk assessment perspective. ACS Chem. Health Saf..

[2] Cyranoski D. (2020). Profile of a killer: The complex biology powering the coronavirus pandemic. Nature.

[3] Hou Y.J., Okuda K., Edwards C.E., Martinez D.R., Asakura T., Dinnon K.H., Kato T., Lee R.E., Yount B.L., Mascenik T.M. (2020). Sars-CoV-2 reverse genetics reveals a variable infection gradient in the respiratory tract. Cell.

[4] Li W., Li M., Ou G. (2020). COVID-19, cilia, and smell. FEBS J..

[5] World Health Organization (2020). Laboratory Testing for Coronavirus Disease (COVID-19) in Suspected Human Cases: Interim Guidance. https://apps.who.int/iris/handle/10665/331501.

[6] Cheng M.P., Yansouni C.P., Basta N.E., Desjardins M., Kanjilal S., Paquette K., Caya C., Semret M., Quach C., Libman M. (2020). Serodiagnostic testing for severe acute respiratory syndrome-related coronavirus 2: A narrative review. Ann. Intern. Med..

[7] Hellou M.M., Górska A., Mazzaferri F., Cremonini E., Gentilotti E., De Nardo P., Poran I., Leeflang M.M., Tacconelli E., Paul M. (2021). Nucleic acid amplification tests on respiratory samples for the diagnosis of coronavirus infections: A systematic review and meta-analysis. Clin. Microbiol. Infect..

[8] Corman V.M., Landt O., Kaiser M., Molenkamp R., Meijer A., Chu D.K., Bleicker T., Brünink S., Schneider J., Schmidt M.L. (2020). Detection of 2019 novel coronavirus (2019-nCoV) by real-time RT-PCR. Detection of 2019 novel coronavirus (2019-nCoV) by real-time RT-PCR. Euro Surveill.

[9] Tosif S., Lee L.Y., Nguyen J., Overmars I., Selman C., Grobler A.C., McMinn A., Waller G., McNab S., Jarvis T. (2023). A novel anterior nasal swab to detect respiratory viruses: A prospective study of diagnostic accuracy. BMC Pediatr..

[10] Lee R.A., Herigon J.C., Benedetti A., Pollock N.R., Denkinger C.M. (2021). Performance of Saliva, Oropharyngeal Swabs, and Nasal Swabs for SARS-CoV-2 Molecular Detection: A Systematic Review and Meta-analysis. J. Clin. Microbiol..

[11] Tsang N.N.Y., So H.C., Ng K.Y., Cowling B.J., Leung G.M., Ip D.K.M. (2021). Diagnostic performance of different sampling approaches for SARS-CoV-2 RT-PCR testing: A systematic review and meta-analysis. Lancet.

[12] Gadenstaetter A.J., Mayer C.D., Landegger L.D. (2021). Nasopharyngeal versus nasal swabs for detection of SARS-CoV-2: A systematic review. Rhinology.

